# Functional shoulder radiography with use of a dynamic flat panel detector

**DOI:** 10.1007/s12194-014-0257-2

**Published:** 2014-02-11

**Authors:** Keita Sakuda, Shigeru Sanada, Rie Tanaka, Katsuhiko Kitaoka, Norio Hayashi, Yukihiro Matsuura

**Affiliations:** 1 Division of Health Sciences, Radiology, Graduate School of Medical Science, Kanazawa University, 5-11-80 Kodatsuno, Kanazawa, Ishikawa, 920-0942 Japan; 2 Department of Radiological Technology, Kanazawa University Hospital, 13-1 Takaramachi, Kanazawa, Ishikawa, 920-8641 Japan; 3 Department of Radiological Technology, School of Health Sciences, College of Medical, Pharmaceutical and Health Sciences, Kanazawa University, 5-11-80 Kodatsuno, Kanazawa, Ishikawa, 920-0942 Japan; 4 Department of Orthopedic Surgery, Koujinkai Kijima Hospital, 41-1 Matsuderamachi, Kanazawa, Ishikawa, 920-0011 Japan; 5 School of Radiological Technology, Gunma Prefectural College of Health Science, 328-1 Kamiokimachi, Maebashi, Gunma, 371-0052 Japan

**Keywords:** Shoulder joint, Dynamic flat panel detector, Scapulothoracic angle, Scapulohumeral rhythm, Ratio of G-angle to S-angle (GSR)

## Abstract

Our purpose in this study was to develop a functional form of radiography and to perform a quantitative analysis for the shoulder joint using a dynamic flat panel detector (FPD) system. We obtained dynamic images at a rate of 3.75 frames per second (fps) using an FPD system. Three patients and 5 healthy controls were studied with a clinically established frontal projection, with abduction of the arms. The arm angle, glenohumeral angle (G-angle), and scapulothoracic angle (S-angle) were measured on dynamic images. The ratio of the G-angle to the S-angle (GSR) was also evaluated quantitatively. In normal subjects, the G-angle and S-angle changed gradually along with the arm angle. The G-angle was approximately twice as large as the S-angle, resulting in a GSR of 2 throughout the abduction of the shoulder. Changes in G-angle and S-angle tended to be irregular in patients with shoulder disorders. The GSR of the thoracic outlet syndrome, recurrent dislocation of the shoulder joint, and anterior serratus muscle paralysis were 3–7.5, 4–9.5, and 3.5–7.5, respectively. The GSR of the anterior serratus muscle paralysis improved to approximately 2 after orthopedic treatment. Our preliminary results indicated that functional radiography by FPD and computer-aided quantitative analysis is useful for diagnosis of some shoulder disorders, such as the thoracic outlet syndrome, recurrent dislocation of the shoulder joint, and anterior serratus muscle paralysis. The technique and procedures described comprise a simple, functional shoulder radiographic method for evaluation of the therapeutic effects of surgery and/or rehabilitation.

## Introduction

The shoulder joint, i.e., the scapulohumeral joint, is composed of several complex structures, including the scapula, humerus, supraspinatus muscle, musculus infraspinatus, teres minor muscle, and subscapularis muscle. The scapulohumeral joint involves a humeral head joined onto the shallow glenoid fossa of the scapula and has the largest range of motion, with the greatest instability, among the human jointed structures.

Glenohumeral joint disorders are caused by excessive exercise, aging, and trauma; affected individuals suffer from pain during movements. In general, these disorders are characterized by various abnormal morphologic and functional findings. For example, the motion of the shoulder blade is decreased in patients with shoulder joint disorders such as repetitious dislocation of the shoulder. The function of the shoulder joint can be evaluated by measurement of the range of shoulder motion and is very useful for orthopedic surgery evaluation [1–3].

In general, conventional radiography, computed tomography (CT), and magnetic resonance imaging (MRI) are performed in clinical practice for evaluation of the shoulder joint during rest. However, these approaches do not provide functional diagnostic information under load, which is essential for planning of a treatment strategy and, for therapy evaluation. For evaluation of shoulder movement, some reports have described the analysis of the dynamic states of the shoulder joint by use of CT, MRI, or ultrasound (US). Inman et al. [4] reported functional shoulder radiography during upward rotation by use of the X-ray TV system. However, this has not come into practical use because of a small field of view (FOV) and low image quality, which could not reveal the accurate form and location of the scapula acetabula. For solving the problem, MRI [5], cinematography [6], goniometry [7–9], and 3-dimensional tracking systems [10–13] have been commonly used in shoulder kinetic study. However, these imaging techniques are not implemented in clinical practice because of limitations such as high radiation doses in CT, complex and time-consuming procedures in MRI, and a small FOV in US [14–16]. In addition, there are occasionally some errors in indirect measurement, such as goniometry and 3D tracking systems.

Recent advances in X-ray detection and digital image post-processing technologies have facilitated the performance of sequential radiography with extremely low radiation doses [17–19]. Thus, we attempted to develop functional shoulder radiography using a dynamic flat panel detector (FPD) with extremely low radiation doses for evaluating the shoulder joint.

## Materials and methods

### Subjects

Approval for the study was obtained from our institutional review board. The subjects gave written informed consent for participation in this study. Clinical cases included five healthy cases (21–22 years old; mean 21.6 years; males:females = 5:0) and three patients with shoulder joint disorders (17–19 years old; mean 18.0 years; males:females = 2:1). Abnormal cases were diagnosed as having decreased movement of a shoulder blade and being seen by an orthopedist based on clinical incidence.

### Image acquisition

Imaging with a fluoroscopic system with a dynamic FPD (Sonial Vision Safire II; Shimadzu) and X-ray tube (CIRCLEX J type 0.4/0.7 JG326D-265AT) was performed under the following imaging conditions : 90 kV, 200 mA, 10 ms, source–detector distance (SDD) 150 cm, 3.75 frames/s, and FOV of 38 × 38 cm. The matrix size of an acquisition picture was 1440 × 1440 pixels, the pixel size was 0.26 mm, and the output was in a 16-bit gray scale.

**Fig. 1:**
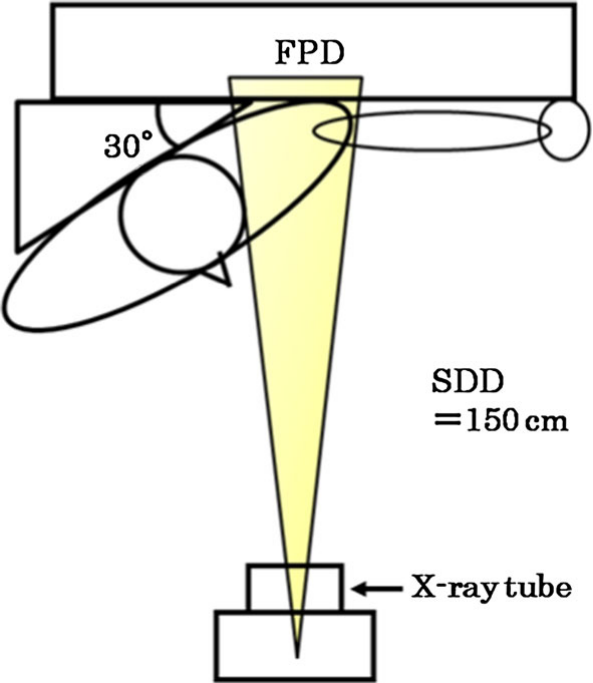
Geometry of the shoulder joint dynamic examination. *SDD* source-detector distance, *FPD* flat panel detector

**Fig. 2:**
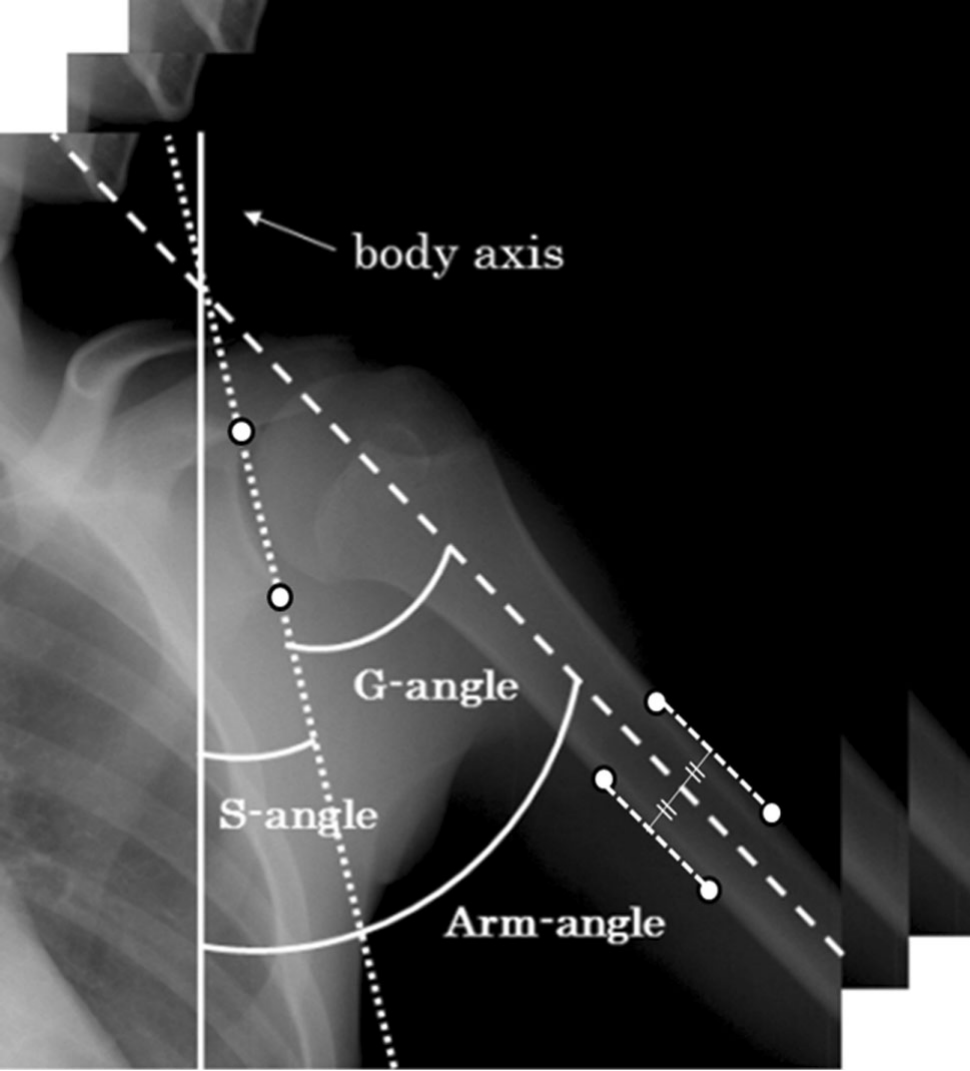
Measurement angles. The *white circles* are the points manually determined by clicking on images. The *solid and broken lines* show the body axis and the *center line* of the humerus, respectively. The *dot line* indicates the line that connects the upper and lower extremities of the shoulder blade acetabular angle

Imaging was performed with the established frontal projection with abduction of the arms, and 15 images were obtained in 4 s (Fig. 1). The total radiation dose was almost equivalent to that of two projections of conventional shoulder imaging (0.4 mGy). For improved reproducibility of the kinetic test, the patients were given sufficient explanations regarding the procedure and how they should participate before actual imaging. Moreover, the spine was included in the FOV to rectify the inclination of the body axis by abduction (Fig. 2).

### Image analysis

#### Angle analysis

We used in-house software (development environment: CodeGear C++ Builder 2008) to analyze the images. The software has a graphic user interface and allows easy and quick angle measurement.

The axis tilt of the body was determined based on the spine and was corrected manually parallel with the y-axis to correct the inclination of the subject’s body axis by affine transform. Figure 2 represents an angle measured in this study. The angles were determined based on the six points (Fig. 2), humerus boundaries, and the upper and lower extremities of the shoulder blade acetabular angles. These points were determined manually by clicking on each frame. All subsequent procedures were performed automatically, as follows:①The center line of the humerus was defined as the middle line of the humerus boundaries in the long-axis direction, and the angle formed by the body axis was calculated automatically as the arm angle. The scapulothoracic angle (S-angle) was defined as the angle formed by the body axis and the line that connected two points, the upper and lower extremities of the shoulder blade acetabular angle. The glenohumeral angle (G-angle) was calculated automatically by subtraction of the arm angle from the scapulothoracic angle. The G-angle and S-angle were measured continuously for each arm angle. In case of body motion during imaging, the inclined spine was compensated for automatically by shifting and rotating of the images on the basis of the body axis before the angle measurements. The measurements were performed in all patients by use of our software. The measurement time per patient was 20 min.

#### Glenohumeral angle:scapulothoracic angle ratio

The normal shoulder joint has a “scapulohumeral rhythm”, which means that the shoulder turns outward and maintains the ratio of the S-angle and the G-angle at 1:2 after the arm angle reaches approximately 60° [4, 20]. For validation of the scapulohumeral rhythm, we investigated the relationship between the arm angle and the G-angle:S-angle ratio (GSR), which was calculated by dividing of the G-angle by the S-angle. We measured the G-angle and S-angle three times per frame in each case, and the average values were used for the calculation of the GSR. It has been reported that normal subjects would maintain a GSR of 2 above an arm angle of approximately 60°, and the GSR would be useful for evaluation of arm angles ≥40° [21]. Thus, in this study, the relationship between the GSR and the arm angle was plotted as line graphs. The results were compared between the right and left shoulders in each individual.

## Results

### Normal controls

**Fig. 3:**
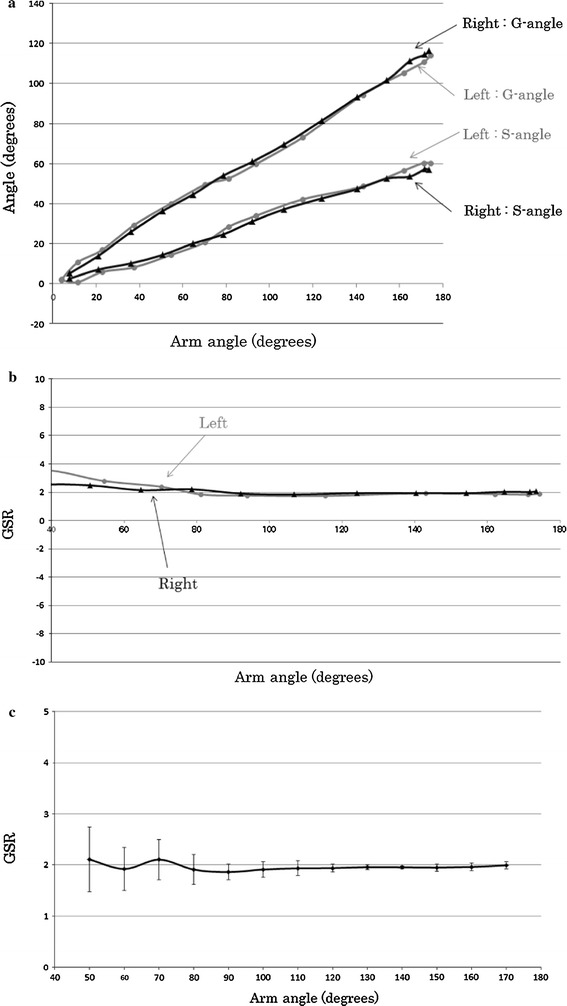
Relationship between G-angle and S-angle (**a**) and GSR (**b**) according to arm angle in a normal control (22-year-old man, normal control) and the average GSR (**c**) obtained from normal controls. *Error bars* show standard deviation for five normal controls and the average GSR (**c**) obtained from normal controls

The kinetic changes in the shoulder joint vary between individuals. Therefore, we analyzed bilateral differences in each subject. Figure 3 shows the analysis result in a normal control. The results were the average values obtained from three measurements of one normal case. The standard deviation of three measurements was <0.3°. The results indicate that the G-angle and S-angle increased gradually according to the increase in the arm angle. The right and left shoulders showed the same movement and resulted in almost the same G-angle and S-angle for each arm angle. The G-angle was approximately twice as large as the S-angle, resulting in a GSR of 2 throughout the abduction of the shoulder. In particular, the GSR was steady after the arm angle increased to ≥60°. These findings were found in all five normal controls. The standard deviation of five normal controls was <0.6° (Fig. 3c).

### Thoracic outlet syndrome

**Fig. 4:**
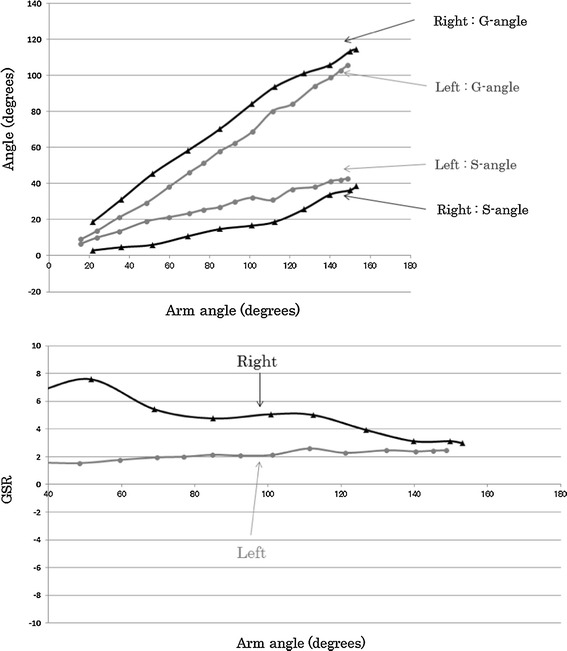
Relationship between G-angle and S-angle (*upper*) and GSR (*lower*) according to arm angle (17-year-old man with thoracic outlet syndrome)

Figure 4 shows the results for a baseball pitcher who had a suspected diagnosis of thoracic outlet syndrome in the right shoulder. Our results indicated that the right S-angle was smaller than the left one throughout shoulder abduction. There was a limitation in the scapular range of motion in the right shoulder joint. In addition, the right shoulder had large GSRs, from 3 to 7, and a disturbed scapulohumeral rhythm. In contrast, a normal scapulohumeral rhythm was observed in the left shoulder joint, with a GSR of 2.

### Recurrent dislocation of the shoulder joint

**Fig. 5:**
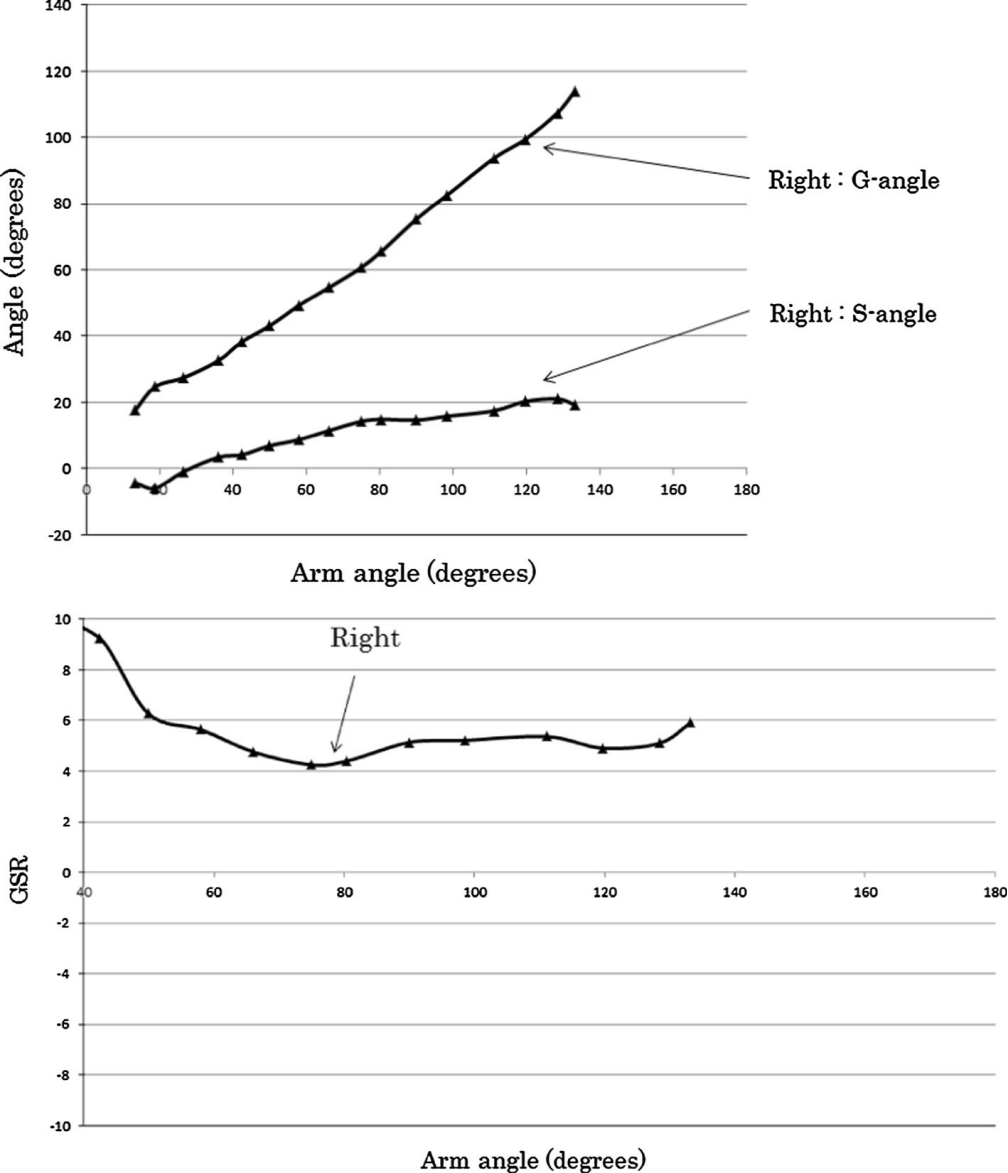
Relationship between G-angle and S-angle (*upper*) and GSR (*lower*) according to arm angle (19-year-old man with recurrent dislocation of the shoulder joint)

Figure 5 shows the results in a patient with repetitious dislocation of the shoulder. Imaging was performed only in the right shoulder because the upper left arm remained fixed after surgery for repair of a bone fracture. In this patient, the S-angle was approximately 20° even at the maximum abduction, which was significantly smaller than that in normal subjects, indicating approximately 60° from an arm angle of approximately 40°. The results indicated decreased motion of the shoulder blade. Moreover, the abduction angle was only approximately 130°, which was considerably smaller than the normal angle of approximately 170°. The results indicated that the movement of the shoulder joint was limited by the impairments. In addition, the GSR ranged from 4 to 6, which was larger than the normal GSR of 2 at an arm angle of ≥60°. The results provided a better understanding of abnormal scapulohumeral rhythm (Fig. 5).

### Long thoracic nerve lesion (paralysis of the serratus anterior muscle)

**Fig. 6:**
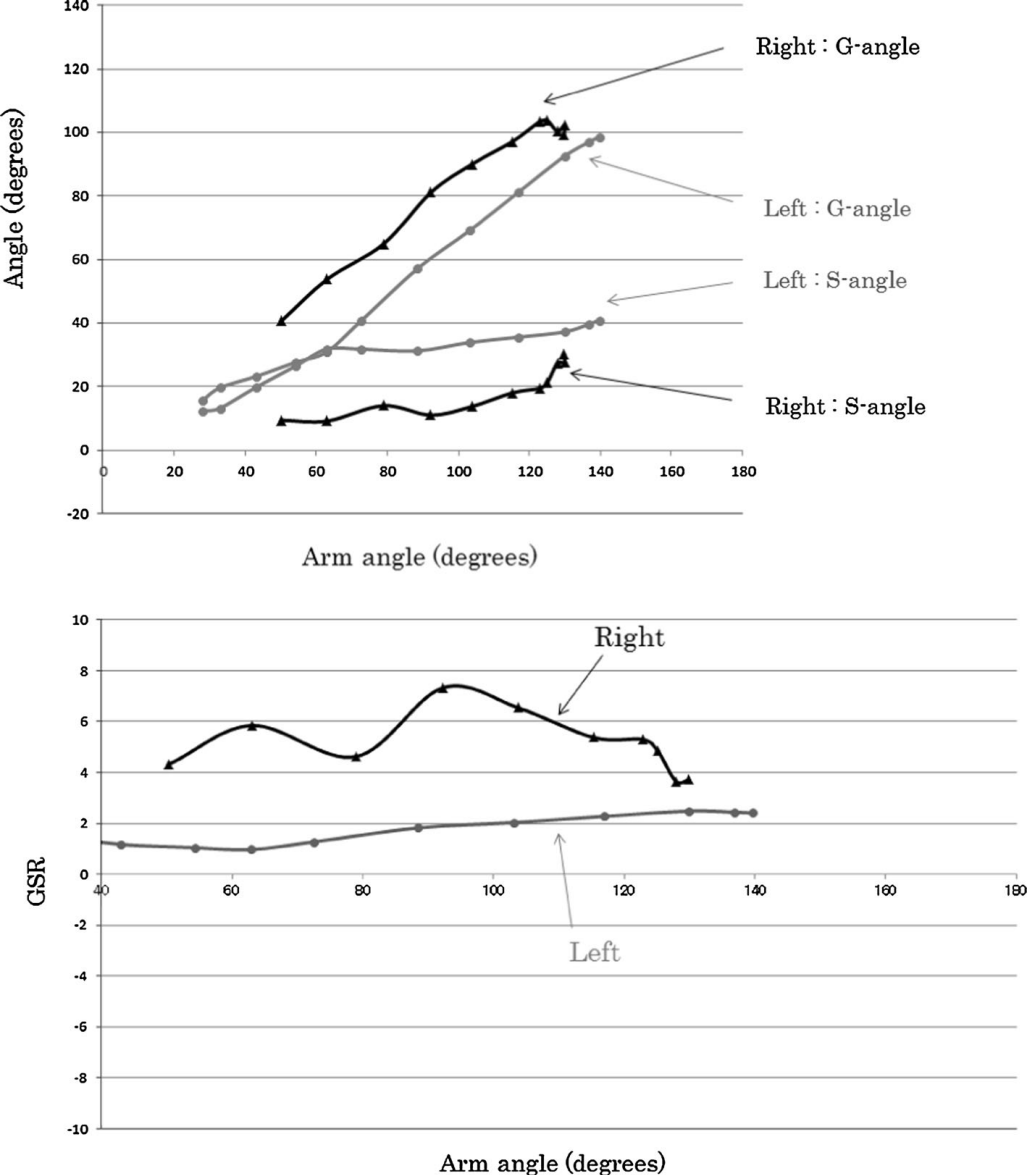
Relationship between G-angle and S-angle (*upper*) and GSR (*lower*) according to arm angle [18-year-old woman with a long thoracic nerve lesion (paralysis of the serratus anterior muscle)]

Figure 6 shows the results for a patient with serratus anterior muscle paralysis due to weight lifting, which led us to suspect a long thoracic nerve lesion. The S-angle in the abnormal right shoulder was smaller than that in the normal left shoulder, and the maximum abduction angle was also decreased. The left shoulder maintained a GSR of 2, whereas the right shoulder GSR ranged from 3 to 7 with a large variation. The severe condition improved after treatment, and the GSR decreased to approximately 2–4. Our results were very useful for quantitative evaluation of the shoulder joint function.

**Fig. 7:**
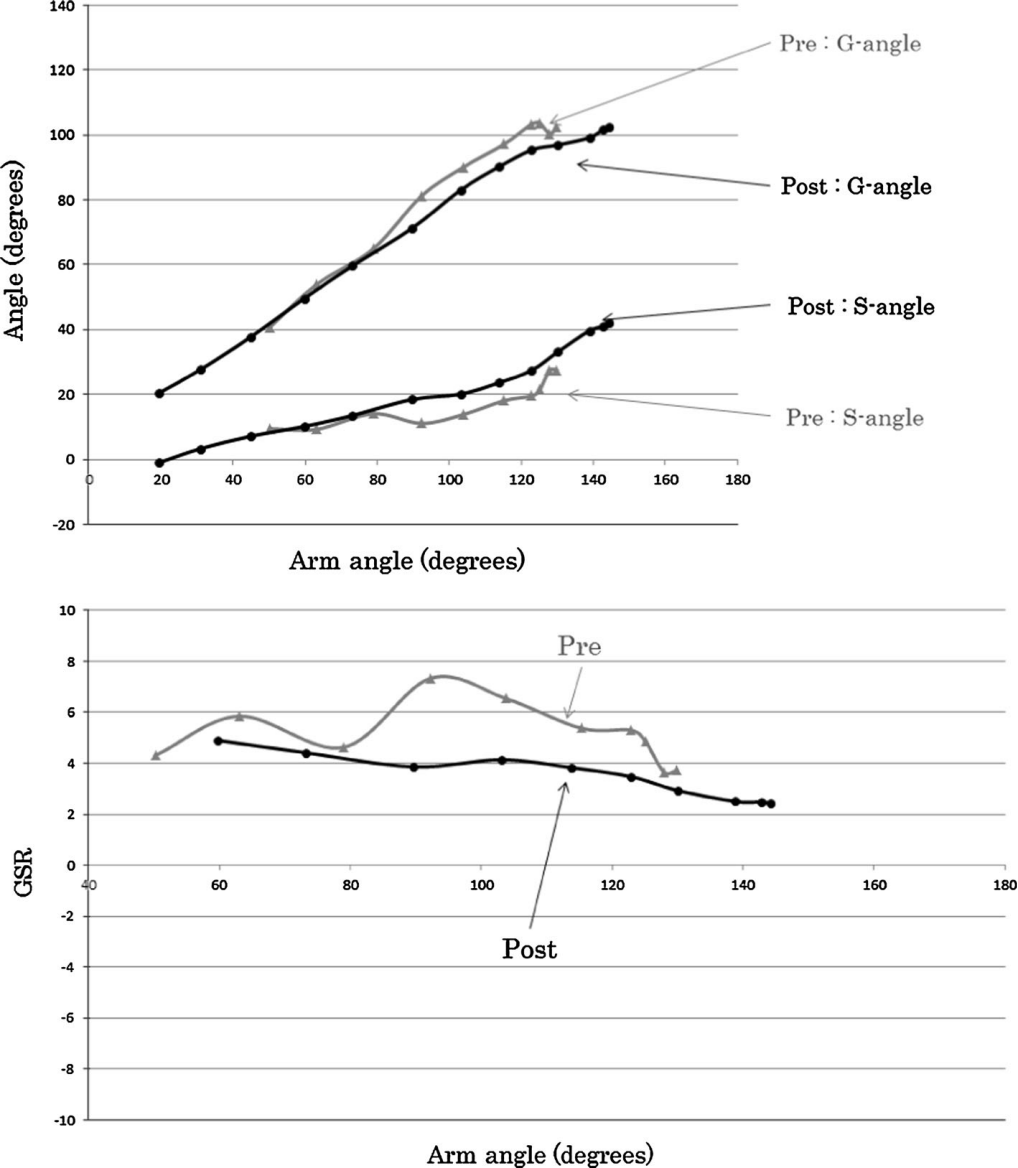
Relationship between G-angle and S-angle (*upper*) and GSR (*lower*) according to arm angle [18-year-old woman with a long thoracic nerve lesion (paralysis of the serratus anterior muscle)], observation of the right shoulder joint

In the follow-up examinations after 1 month, the scapula had a smoother and larger movement (Fig. 7). The maximum abduction angle was improved significantly compared to the pre-rehabilitative measurements. The GSR was nearly 2, revealing an improved scapulohumeral rhythm.

## Discussion

Functional shoulder radiography by use of a dynamic FPD is proposed in this study. Sequential images with a large FOV allow us to observe the movement of a shoulder joint and scapula with an extremely low radiation dose. In addition, the dynamic analysis provided the relationship between the scapula and humerus during abduction.

In normal controls, our results were supported by a normal scapulohumeral rhythm, showing a GSR of 2 above an arm angle of abduction of 60°. These findings were observed in all normal controls in our study. Inman et al. [4] reported that the scapulohumeral rhythm was 2:1, which was the ratio between glenohumeral angle elevation and scapulothoracic angle obtained by use of radiography during upward rotation. Our results are supported by their report. In contrast, in the case of any thoracic outlet syndrome, shoulder instability arthropathy, and long thoracic nerve injury, they showed GSR values of 2 and much larger. Furthermore, in a patient with a follow-up examination, we confirmed the improvement of shoulder joint motion, as shown in Fig. 7. These results indicate that functional shoulder radiography combined with computer analysis could detect functional abnormalities. The present method would be useful for evaluation of the scapulohumeral rhythm, especially in cases when the humerus arm looks like normal movement, but the actual movement of the scapula is limited.

Functional shoulder radiography with the FPD, providing direct observation of the shoulder blade, resulted in exact assessments of shoulder kinetics. In terms of accuracy of the analysis, the standard deviation of the average GSR obtained by three analysts was <0.1° due to clear depiction of the scapula acetabula. Although still requiring several manual operations, high image quality and resolution allow the accurate and reproducible determination of measurement points. Furthermore, the total patient dose is almost the same as that in conventional shoulder radiography in two projections. The present method would be a promising means of functional imaging for use in daily clinical practice.

For clinical implementation, there are still some problems to be resolved. One of the top priorities is shortening the processing time of the angle measurements. In this study, the angle measurement was performed by use of semi-automatic software that we developed. The software provided the G-angle and S-angle automatically just by clicking on six points on the images (Fig. 2). However, measurements are needed for all of the frames and resulted in troublesome tasks for clinicians. Manual correlation of the body axis is also required before the angle measurements. For use in daily clinical practice, further studies are required for simplifying the procedures by automatic extraction of the humerus, the scapula acetabular rim, and spine.

## Conclusion

Functional shoulder radiography by use of a dynamic FPD with a low radiation dose allowed us to evaluate joint motion. The software we developed was helpful for quantitative measurement of the shoulder joints. The technique and procedures described comprise a simple, low radiation dose, and a cost-effective, functional shoulder radiographic method for evaluation of the therapeutic effects of surgery and/or rehabilitation.
